# Effect of Different Levels of Peep on Oxygenation during Non-Invasive Ventilation in Patients Submitted to CABG Surgery: Randomized Clinical Trial

**DOI:** 10.21470/1678-9741-2016-0038

**Published:** 2017

**Authors:** André Luiz Lisboa Cordeiro, Caroline Aparecida Gruska, Pâmella Ysla, Amanda Queiroz, Sarah Carvalho de Oliveira Nogueira, Maria Clara Leite, Bruno Freitas, André Raimundo Guimarães

**Affiliations:** 1 Escola Bahiana de Medicina e Saúde Pública (EBMSP), Salvador, BA, Brazil; 2 Faculdade Nobre (FAN), Feira de Santana, BA, Brazil; 3 Instituto Nobre de Cardiologia (INCARDIO), Feira de Santana, BA, Brazil; 4 Santa Casa de Misericórdia de Feira de Santana, Feira de Santana, BA, Brazil

**Keywords:** Oxygenation, Noninvasive Ventilation, Cardiovascular Surgical Procedures, Coronary Artery Bypass

## Abstract

**Introduction:**

During and after coronary artery bypass grafting, a decline in multifactor lung function is observed. Due to this fact, some patients may benefit from non-invasive ventilation after extubation targeting lung expansion and consequently improved oxygenation.

**Objective:**

To test the hypothesis that higher levels of positive end expiration pressure during non-invasive ventilation improves oxygenation in patients undergoing coronary artery bypass grafting.

**Methods:**

A randomized clinical trial was conducted at Instituto Nobre de Cardiologia in Feira de Santana. On the first day after surgery, the patients were randomized: Group PEEP 10, Group PEEP 12 and Group PEEP 15 who underwent non-invasive ventilation with positive end expiration pressure level. All patients were submitted to analysis blood pressure oxygen (PaO_2_), arterial oxygen saturation (SaO_2_) and oxygenation index (PaO_2_/FiO_2_).

**Results:**

Thirty patients were analyzed, 10 in each group, with 63.3% men with a mean age of 61.1±12.2 years. Mean pulmonary expansion pre-therapy PaO_2_ was generally 121.9±21.6 to 136.1±17.6 without statistical significance in the evaluation among the groups. This was also present in PaO_2_/FiO_2_ and SaO_2_. Statistical significance was only present in pre and post PEEP 15 when assessing the PaO_2_ and SaO_2_ (*P*=0.02).

**Conclusion:**

Based on the findings of this study, non-invasive ventilation with PEEP 15 represented an improvement in oxygenation levels of patients undergoing coronary artery bypass grafting.

**Table:** 

Abbreviations, acronyms & symbols				
BMI	= Body mass index		IPPB	= Intermittent positive pressure breathing
CABG	= Coronary artery bypass grafting		LET	= Lung expansion therapy
COPD	= Chronic obstructive pulmonary disease		MEP	= Maximal expiratory pressure
CPB	= Cardiopulmonary bypass		MIP	= Maximal inspiratory pressure
CVD	= Cardiovascular diseases		MV	= Mechanical ventilation
ETT	= Endotracheal tube		NIV	= Non-invasive ventilation
ICU	= Intensive care unit		PaO_2_	= Blood pressure oxygen
IMV	= Invasive mechanical ventilation		PEEP	= Positive end expiration pressure
			SaO_2_	= Arterial oxygen saturation

## INTRODUCTION

Cardiovascular diseases (CVD) are among the leading causes of death in developed countries and have been increasing in epidemic form in emerging countries^[1]^. Cardiac surgery are highly invasive procedures, large, indicated and used for treatment in the medium/long-term CVD and postoperative complication rates are mostly for pulmonary complications^[2]^.

Coronary artery bypass grafting (CABG) generates an effect on lung function with consequent decrease in functional residual capacity, and providing the appearance in atelectasis in the postoperative period and pulmonary shunt, causing the patient evolve with hypoxia and imbalance in the relationship ventilation/oxygenation^[3,4]^.

Noninvasive ventilation (NIV) have been one of physiotherapy methods used during the immediate postoperative CABG, aiming to expand the alveolar areas and improve gas exchange and reduce respiratory distress, promoting decreased work of breathing and preventing involvement of atelectasis^[5]^.

Many factors are known to contribute or not to the success of NIV, in which the patient's age, comorbidities presented by the patient (hypertension and diabetes mellitus), cardiopulmonary bypass (CPB) time to that was submitted, the number of bypasses that were performed during the CABG, the amount of placed drains, time of mechanical ventilation (MV) in the postoperative period, the positive end expiration pressure (PEEP) that was used for recruitment during the NIV protocol, among others.

Considering the scientific reports of benefits to the patients using the correct NIV protocol and few data reported in the literature with reference to the factors that affect the success of treatment, this study aims to test the hypothesis that higher levels of PEEP during NIV, improve oxygenation and gas exchange in patients undergoing cardiac surgery CABG.

## METHODS

A randomized clinical trial was conducted with patients who underwent cardiac surgery at Instituto Nobre de Cardiologia/Santa Casa de Misericordia in Feira de Santana - Bahia. Thirty patients were analyzed in the period from February to September 2016. The inclusion criteria had patients undergoing CABG via sternotomy and CPB, both genders and aged above 18 years. Exclusion individuals with chronic obstructive pulmonary disease (COPD), emergency surgery, heart surgery, hemodynamic instability at the time of completion of the NIV, patients who remained in the invasive mechanical ventilation (IMV) for a period longer than ten hours, uncontrolled arrhythmias, lack of collaboration and/or understanding of the procedure, claustrophobia, nausea/vomiting or any other contraindications of NIV and patients who did not accept to sign the consent form.

After inclusion in the study, all patients had their sociodemographic and clinical characteristics evaluated at the time of admission to the intensive care unit (ICU). Upon arrival to the ICU, all of them were in IMV via endotracheal tube (ETT). All handling in the immediate postoperative period was performed by the staff on duty, using routine criteria of the unit without any influence of researchers, worth mentioning that the VMI interruption decision also followed this routine.

On the first day after surgery, the patients were randomly assigned by lot into three groups: Group 10 (G10) who carried out the NIV with a PEEP of 10 cmH_2_0; Group 12 (G12) NIV with PEEP of 12 cm H_2_O; and Group 15 (G15) who underwent NIV with PEEP 15 cm H_2_O.

All patients underwent collection of arterial blood sample was analyzed on a gas meter to check the values of blood pressure of oxygen (PaO_2_), arterial oxygen saturation (SaO_2_) and oxygenation index (PaO_2_/FiO_2_). After the first collection, vital signs were measured (heart rate, systolic and diastolic blood pressure, respiratory rate and oxygen saturation) were monitored throughout the period of NIV.

Patient monitoring underwent a lung expansion therapy (LET) in NIV (Mode Pressure Support (PS) plus PEEP, PS aiming at a tidal volume of 6 to 8 ml/kg, PEEP group randomized and FiO_2_ 40%) in Serbo-S ventilator (Dräger Medical, Lübeck, Germany) for 40 minutes via facial mask with PEEP values. Immediately at the end of therapy and one later, new hemogasometry were collected for comparison to baseline. All patients were extubated in the ICU without extubation in the surgical center and none of the patients studied were reintubated during the collection period.

### Statistical Analysis

SPSS 20.0 software was used. To test the distribution of the sample, the Shapiro-Wilk test was used. Data were expressed as mean and standard deviation or median and interquartile range. For the analysis of the characteristics and differences among the groups, the Kruskal-Wallis test was used. To analyze the before and after NIV within each group, the Wilcoxon test was used. It is considered as significant an alpha of 5%. All research was conducted in accordance with ethical standards being released by the Research Ethics Committee of Faculdade Nobre under the number 51209815.7.0000.5654.

## RESULTS

**Fig. 1:**
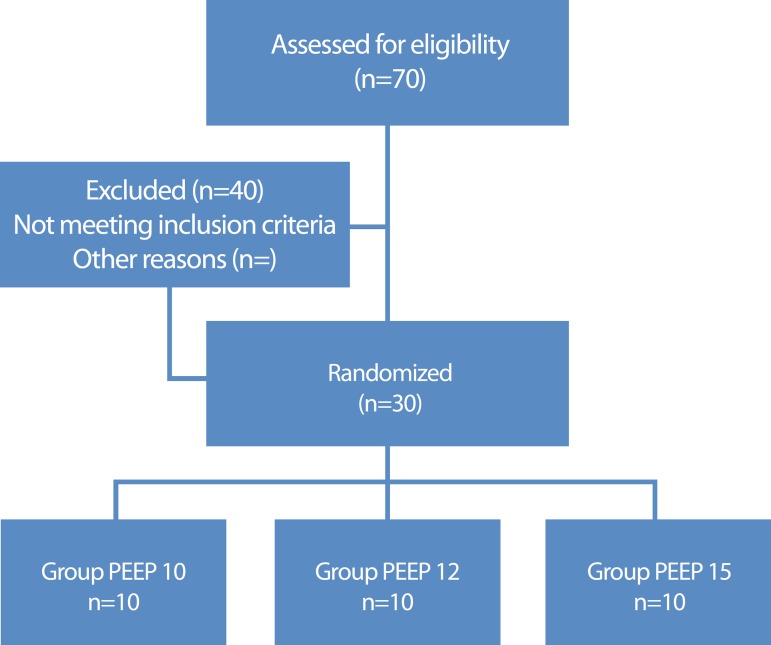
Patient selection flowchart.

Between January and September 2016, 70 patients were admitted for cardiac surgery at the Instituto Nobre de Cardiologia, 40 of whom were excluded due to valve or combined surgery (39 patients) and one due to hemodynamic instability before the fifth minute of NIV. Figure 1 demonstrates the flow for patient selection. Including 30 patients, for convenience, 63.3% men, with mean age of 61.1±12.2 years. The groups were homogeneous in relation to the demographic and surgical characteristics. Tables 1 and 2 present these characteristics of the patients included in the study.

**Table 1:** Demographic characteristics of patients undergoing myocardial revascularization.

Variable	PEEP 10 n=10	PEEP 12 n=10	PEEP 15 n=10	*P*
Gender				0.87^a^
Male	7 (70%)	6 (60%)	6 (60%)	
Female	3 (30%)	4 (40%)	4 (40%)	
Age (years)	57.5±20	61.7±7.3	64.2±9.2	0.62^b^
BMI	23.7±4.1	23.1±3.2	22.9±2.5	0.88^b^
Comorbidities				
Hypertension	4 (40%)	6 (60%)	2 (20%)	0.2^a^
DM	10 (100%)	10 (100%)	5 (50%)	0.003^a^
Dyslipidemia	4 (40%)	4 (40%)	3 (30%)	0.87^a^
Smoking	__	2 (20%)	1 (10%)	0.34^a^
AMI	__	1 (10%)	2 (20%)	0.34^a^

a Chi-square test;

b Kruskal-Wallis test; PEEP=positive end expiratory pressure; BMI=body mass index; DM=diabetes melitus; AMI=acute myocardial infarction

**Table 2:** Clinical and surgical characteristics of patients undergoing myocardial revascularization.

Variable	PEEP 10	PEEP 12	PEEP 15	*P*^a^
Extracorporeal circulation (min)	68.9±15.6	76.1±23.6	74.3±22.1	0.73
Bypass numbers	3 (1.75-3)	3 (1.75-3)	3 (2-3)	0.94
Number of drains	2 (2-2)	2 (1.75-2)	2 (2-2.25)	0.14

a Kruskal-Wallis test; PEEP=positive end expiratory pressure

Mean pulmonary arterial pressure was generally 121.9±21.6 to 136.1±17.6 without statistical significance in the evaluation among the groups. It was also present in relation to oxygenation index and arterial oxygen saturation. Statistical significance was only present in pre and post PEEP 15 when assessing the PaO_2_ and SaO_2_. Table 3 shows the behavior of these values in different groups.

**Table 3:** Analysis of oxygenation in patients undergoing cardiac surgery.

Variable	PEEP 10	PEEP 12	PEEP 15	*P*^a^
PaO_2_ (mmHg)				
Before	124.8±16.5	128.1±28.8	112.9±19.5	0.26
After	131.3±16.8	137.6±25	139.4±11	0.53
*P*^b^	0.33	0.11	0.02	
Oxygenation Index (mmHg)				
Before	312±41.3	320.4±72	282.3±48.8	0.29
After	328.3±42.1	343.9±62.6	348.5±27.6	0.6
*P*^b^	0.33	0.11	0.02	
Arterial Oxygen Saturation (%)				
Before	97±2	96.6±1.5	96.3±2.5	0.71
After	96.9±1	97.2±1.3	96.1±3.3	0.52
*P*^b^	0.76	0.21	0.86	

a Kruskal-Wallis test;

b Wilcoxon test; PEEP=positive end expiratory pressure; PaO2=oxygen blood pressure

An initial concern was present in relation to hemodynamic forward behavior to high levels of PEEP. Table 4 shows that no variable has tabled an amendment meant statistically significant. In addition, only one adverse event was associated with increased PEEP and the amount of pressure was 10 cm H_2_O, which led to the exclusion of the research patient.

**Table 4:** Hemodynamic impact of lung re-expansion therapy in patients undergoing cardiac surgery.

Variable	PEEP 10	PEEP 12	PEEP 15	*P*^a^
HR (bpm)				
Before	97.6±12.6	92.3±13.9	86.6±15.1	0.21
After	92±13.2	90±14.6	87.1±13.3	0.7
*P*^b^	0.15	0.72	0.93	
SBP (mmHg)				
Before	119.7±10.6	121.4±11.6	114.8±9.2	0.31
After	118±12.8	123.3±14.9	118.5±17.5	0.60
*P*	0.72	0.67	0.58	
DBP (mmHg)				
Before	62.8±9	60.7±8.4	62.9±11.7	0.84
After	61.4±10.3	60.2±7.8	67±18.9	0.74
*P*	0.35	0.84	0.24	
MAP (mmHg)				
Before	80.2±7.7	79.9±8.6	77.7±8.5	0.92
After	79±8.8	79.4±7.1	84.2±16.9	0.76
*P*	0.31	0.99	0.16	

a Kruskal-Wallis test;

b Wilcoxon test; PEEP=positive end expiratory pressure; HR=heart rate; SBP=systolic blood pressure; DBP=diastolic blood pressure; MAP=mean arterial pressure

## DISCUSSION

Based on the findings of this study, the value of overpressure of PEEP does not influence gas exchange during the NIV in patients on the first day after cardiac surgery.

Barbas et al.^[6]^, bring as a recommendation to use NIV in postoperative abdominal and elective thoracic surgery, for the treatment of acute respiratory insufficiency and in order to reduce atelectasis and need for endotracheal intubation, improve gas exchange and reduce respiratory work.

The success and/or failure of NIV therapy in postoperative CABG may be associated with characteristics such as gender and body fat distribution. The study by Gonçalves et al.^[7]^ shows that each unit added to the individual's body mass index decreased by -1.908 value of maximal inspiratory pressure (MIP) in men and -1.703 in women. Regarding maximal expiratory pressure (MEP), each unit added in BMI increased 0.184 its value in men and 0.651 in women. In view of this fact and analyzing the results presented in this study, it is noted that the variable BMI, according to Table 1, did not contribute effectively to change the effectiveness, and the patients were within the normal classification.

In 2013, a published article aimed to compare the application of NIV with usual care in this patient profile. Researchers have shown that the use venting reduced postoperative complication rate as well as the time of stay in the ICU^[8]^.

Lima et al.^[9]^ evaluated 78 patients divided into three groups. Each group performed a lung expansion therapy with different levels of PEEP, and 05, 8:10 cmH_2_O values. They demonstrated that the use of different levels of PEEP did not affect gas exchange in patients undergoing CABG. It is noteworthy that this therapy was performed pre-extubation endotracheal and in this study LET was conducted during the NIV. In 2013, Borges et al.^[10]^ used the same Lima study^[9]^ seeking to assess the impact on oxygenation and lung mechanics on the finding that higher levels of PEEP increased the lung compliance values and improved oxygenation index.

Already Celebi et al.^[11]^ stated in their research that the NIV associated with alveolar recruitment maneuver promotes improved oxygenation during and after the period of MV. The authors evaluated hundred patients undergoing CABG divided into four groups. This result contradicts the findings of the present study and other authors.

A Chinese group also assessed the impact of NIV in patients for aortic dissection correction. In this study, they used a PEEP 8-10 cm H_2_O and found the impact of therapy on gas exchange in the first and sixth hour, concluding that the NIV positively influences oxygenation in that population^[12]^.

In a national study, 100 patients were evaluated who underwent CABG in order to demonstrate the benefits of NIV after extubation. In this research, the group performed the NIV and was pressurized with a PEEP of 5 cm H_2_O, is below the present study, for 30 minutes, also different time of the study. They found an improvement with statistical significance (*P*=0.0009), oxygenation of patients who underwent the technique^[13]^. Preisig et al.^[14]^ evaluated the gas exchange and hemodynamic changes in patients undergoing cardiac surgery who had hypoxemia. They found an increase in PaO_2_ and oxygenation index up to three hours after the end of NIV. On the other hand, oxygen saturation was not statistically significant. However, a change was observed in pulmonary capillary wedge pressure which may represent negative hemodynamic impact with PEEP levels of 7 cmH_2_0.

Regarding hemodynamic variables, this study showed that the level of PEEP does not adversely affect blood pressure and heart rate. Similar data were expressed by Lopes et al.^[13]^ where no significant differences in the variables was observed, and the NIV group showed *P*=0.671 (heart rate) and *P*=0.498 (mean arterial pressure). These results corroborate the study of Franco et al.^[15]^ who claim that application NIV preventively in the postoperative period is safe, maintaining stable hemodynamics.

A limitation of this study was the small number of patients in the sample and thus it could not properly assess whether the intervention with different levels of PEEP affect oxygenation. But it is noteworthy that this is the first study to assess different levels of PEEP during intermittent positive pressure breathing (IPPB) in patients undergoing cardiac surgery.

## CONCLUSION

Based on the findings of this study, the value of overpressure of PEEP does not influence gas exchange during the NIV in patients on the first day after cardiac surgery.

**Table:** 

Authors' roles & responsibilities	
ALLC	Drafting the work or revising it critically for important intellectual content; final approval of the version to be published
CAG	Substantial contributions to the conception or design of the work; or the acquisition; final approval of the version to be published
PY	Substantial contributions to the conception or design of the work; final approval of the version to be published
AQ	Substantial contributions to the conception or design of the work; final approval of the version to be published
SCON	Substantial contributions to the conception or design of the work; final approval of the version to be published
MCL	Substantial contributions to the conception or design of the work; final approval of the version to be published
BF	Substantial contributions to the conception or design of the work; final approval of the version to be published
ARG	Substantial contributions to the conception or design of the work; final approval of the version to be published
